# Performance of a New Instrument for the Measurement of Systemic Lupus Erythematosus Disease Activity: The SLE-DAS

**DOI:** 10.3390/medicina59122097

**Published:** 2023-11-29

**Authors:** Malcolm Koo, Ming-Chi Lu

**Affiliations:** 1 Department of Nursing, Tzu Chi University of Science and Technology, Hualien 970302, Taiwan; m.koo@utoronto.ca; 2 Dalla Lana School of Public Health, University of Toronto, Toronto, ON M5T 3M7, Canada; 3 Division of Allergy, Immunology and Rheumatology, Dalin Tzu Chi Hospital, Buddhist Tzu Chi Medical Foundation, Chiayi 622401, Taiwan; 4 School of Medicine, Tzu Chi University, Hualien 970374, Taiwan

**Keywords:** systemic lupus erythematosus, SLEDAI-2K, SLE-DAS, disease activity, measurement scale

## Abstract

Systemic lupus erythematosus (SLE) is a chronic systemic autoimmune disease that affects multiple organ systems and manifests in a relapsing–remitting pattern. Consequently, it is paramount for rheumatologists to assess disease activity, identify flare-ups, and establish treatment goals for patients with SLE. In 2019, the Systemic Lupus Erythematosus Disease Activity Score (SLE-DAS) was introduced as a novel tool for measuring disease activity. This tool refines the parameters of the established SLE Disease Activity Index 2000 (SLEDAI-2K) to enhance the assessment process. This review aims to provide an introduction to the Systemic Lupus Erythematosus Disease Activity Score (SLE-DAS) and summarizes research on its development, its comparison with existing disease activity measures, and its performance in clinical settings. Literature searches on PubMed using the keyword “SLE-DAS” were conducted, covering publications from March 2019 to September 2023. Studies that compared SLE-DAS with other SLE disease activity measurement tools were reviewed. Findings indicated that SLE-DAS consistently performs on par with, and sometimes better than, traditional measures in assessing clinically meaningful changes, patient improvement, disease activity, health-related quality of life, hospitalization rates, and disease flare-ups. The association between SLE-DAS and mortality rates among patients with SLE, however, remains to be further explored. Although SLE-DAS is a promising and potentially effective tool for measuring SLE disease activity, additional research is needed to confirm its effectiveness and broaden its clinical use.

## 1. Introduction to Systemic Lupus Erythematosus (SLE)

Systemic lupus erythematosus (SLE) is a complex, chronic systemic autoimmune disease characterized by the production of autoantibodies against a variety of self-antigens. It can affect multiple bodily systems and organs, including the kidneys, skin, brain, heart, lungs, hematologic system, and musculoskeletal system, leading to widespread inflammation and tissue damage.

Globally, the incidence of newly diagnosed cases of systemic lupus erythematosus (SLE) is estimated at 5.14 per 100,000 person-years, ranging from 1.4 to 15.13, leading to approximately 400,000 new individuals affected by SLE each year. Additionally, the global prevalence of SLE is estimated at 43.7 per 100,000 persons, varying from 15.87 to 108.92, corresponding to approximately 3.41 million people currently living with the disease [1]. The variation in incidence and prevalence can be attributed to differences in population genetics, environmental factors, socioeconomic status, the availability of healthcare, and the comprehensiveness of healthcare records [2].

SLE predominantly affects women, with a female-to-male ratio of 9:1 [1]. The female predominance is even higher during peak childbearing ages. The pathophysiological mechanisms responsible for sexual dimorphism in SLE are still unclear, but cytokine pathways and genetics have been proposed to explain this sexual dimorphism [3].

## 2. Challenges in the Management of SLE

One of the main features of SLE is its unpredictable course, with periods of low or no disease activity (relapsing) alternating with periods of high disease activity (flares). Flares can cause damage to the affected organs and impair the quality of life of patients. In addition, most patients initially present with mild disease activity but may progress to moderate and severe conditions over time [4,5]. Thus, there is a need to measure disease activity to allow clinicians to identify flares early, potentially mitigating organ damage and improving patient outcomes.

While there have been significant improvements in the long-term outcomes for patients with SLE over the past decades, the persistence of increased morbidity and mortality, particularly among young individuals, remains a concern [6]. A meta-analysis of 15 reports involving a total of 26,101 patients with SLE and 4640 deaths revealed that the all-cause standardized mortality ratio (SMR) significantly increased 2.6-fold in patients with SLE. Specifically, the risks of mortality were significantly increased for deaths attributed to renal disease (SMR 4.69, 95% CI 2.36–9.33), cardiovascular disease (SMR 2.25, 95% CI 1.30–3.89), and infection (SMR 4.98, 95% CI 3.88–6.40) [7]. Regular monitoring of disease activity helps in assessing the risk and extent of organ damage, which is critical for tailoring treatment plans.

Recently, there is growing interest in applying the treat-to-target (T2T) approach to SLE treatment, similar to its successful implementation in other rheumatic diseases like rheumatoid arthritis and ankylosing spondylitis. Rheumatologists are considering the adoption of the T2T strategy for SLE treatment. Central to the T2T strategy is the ability to define specific treatment goals and precisely measure disease activity, which are crucial for guiding therapeutic decisions. This approach enables early detection of disease flares and continuous monitoring of treatment efficacy, ensuring timely adjustments to meet set targets. However, further discussions and investigations are needed before T2T can be fully integrated into clinical practice for SLE [8].

## 3. Measurement of Disease Activity in SLE

As outlined above, measuring disease activity in SLE is fundamental to effectively managing this complex autoimmune disease. However, there is still a lack of a single definition or measure of disease activity. This variability in measurement tools reflects the diverse manifestations and complexities of SLE. Consequently, the choice of an assessment tool often depends on the specific clinical context and the aspects of the disease that need to be monitored. 

Various tools have been developed to assess disease activity and organ damage in patients with SLE [9]. These tools include the British Isles Lupus Assessment Group Index (BILAG) [10], the European Consensus Lupus Activity Measurements (ECLAM) [11], the SLICC/ACR Damage Index (SDI) [12], the SLE Disease Activity Index 2000 (SLEDAI-2K) [13], the Safety of Estrogens in Lupus Erythematosus National Assessment (SELENA)-SLEDAI [14], the Systemic Lupus Erythematosus Disease Activity Score (SLE-DAS) [15], and Easy-BILAG [16]. They capture different aspects of disease activity, such as clinical signs and symptoms, laboratory test results, organ involvement, patient-reported outcomes, and physician assessments. 

Among these assessment tools, SLEDAI-2K is commonly used in both clinical practice and basic SLE research. Notably, SLE-DAS, a relatively recent addition, was developed by expanding upon the elements in SLEDAI-2K [15]. This review aims to explore recent studies that have assessed the performance of SLE-DAS in comparison to other SLE-related disease measurements. The focus will be on treatment response, the occurrence of flares, the impact on health-related quality of life (HRQoL), the risk and frequency of hospitalization, disease activity, disease activity during pregnancy, and identification of potential treatment targets for patients with SLE.

## 4. Development and Characteristics of SLE-DAS

SLE-DAS, introduced by Jesus et al. in 2019, is an enhancement to the widely used SLEDAI-2K. This index was developed with two representative longitudinal cohorts of Caucasian patients, comprising 324 individuals in the derivation cohort and 196 in the external validation cohort. It has shown good construct validity and a higher discriminative power for detecting clinically meaningful changes in SLE disease activity compared to its predecessor, SLEDAI-2K. The correlation coefficients between SLE-DAS and SLEDAI-2K were significant in both the derivation and the external validation cohorts at the last follow-up visit (both at r = 0.94). In addition, the predictive value of SLE-DAS in assessing damage accrual over follow-up was better than that of SLEDAI-2K, further demonstrating its criterion validity [15].

One of the key modifications of SLE-DAS is the inclusion of several critical items in evaluating disease activity. These additions include cardiac/pulmonary involvement, gastrointestinal issues, sterile peritonitis, and hemolytic anemia. The index also employs continuous measures for conditions including arthritis, proteinuria, thrombocytopenia, and leucopenia. However, SLE-DAS excludes items including urinary casts, hematuria, pyuria, and fever. Consequently, SLE-DAS comprises a 17-item assessment, a modification from the original 24-item format of SLEDAI-2K [15]. 

Given that SLE-DAS is based on the SLEDAI-2K, positive correlations between the two indices were expected. This was evidenced in a retrospective medical record study of 41 patients with lupus nephritis, where a moderate positive correlation (r = 0.70, *p* < 0.001) was observed at baseline. The correlation was higher at 6-month follow-up, reaching a correlation coefficient of 0.92 (*p* < 0.001) [17]. Moreover, in a cross-sectional study of 333 Taiwanese outpatients with SLE, a significant correlation between SLE-DAS and SLEDAI-2K was also found (Spearman ρ = 0.78; 95% CI 0.71, 0.83; *p* < 0.001) [18]. 

While the scoring process for SLE-DAS can be tedious due to its comprehensive nature, the task can be facilitated using spreadsheet applications such as Microsoft Excel, or through the user-friendly web-based calculator available at http://sle-das.eu/ (accessed on 28 November 2023). These tools allow SLE-DAS to be easier to implement in clinical settings, thereby enhancing its practical utility in the management of SLE.

## 5. Comparison of SLE-DAS and Other SLE Activity Indices

Jesus et al. showed a strong correlation between both SLE-DAS and SLEDAI-2K scores with the Physician Global Assessment (PGA) [15]. When using score changes (Δ) in SLE-DAS of ≥ 1.72 and SLEDAI-2K of ≥ 4 as indicators of clinically meaningful improvement, the researchers found the sensitivity was 82.1% and the specificity was 96.9% for ΔSLE-DAS ≥ 1.72. In contrast, for ΔSLEDAI-2K ≥ 4, the sensitivity was significantly lower at 44.8%, and the specificity remained high at 96.5%. This suggests that SLE-DAS performed better in detecting clinically meaningful changes in SLE compared to SLEDAI-2K. An important factor contributing to this difference is the use of continuous measures for arthritis, proteinuria, thrombocytopenia, and leucopenia in SLE-DAS, which offers a great advantage over the categorical classification of these factors in SLEDAI-2K.

Saraiva et al. investigated flare-ups in 442 patients with SLE who initially presented with low disease activity. These flare-ups were identified based on expert-consensus definitions during follow-up visits. The researchers used various indices for classifying these flare-ups, including the SLE-DAS (Δ ≥ 1.72), classic-SELENA Flare Index (c-SFI), revised-SFI (r-SFI), and SLEDAI-2K (Δ ≥ 4). The study revealed that the sensitivities for detecting SLE flare-ups were 97.1% for SLE-DAS, 88.4% for both c-SFI and r-SFI, and 56.5% for SLEDAI-2K. For specificities, the results were 97.3% for SLE-DAS, 98.1% for c-SFI, 96.8% for r-SFI, and a notably high 99.2% for SLEDAI-2K. Among these four measurements, SLE-DAS exhibited the best discriminative ability in identifying flares, as determined through receiver operating characteristic curve analysis [19]. The study also noted that some patients with SLE might experience flare-ups in only specific domains, such as thrombocytopenia, leukopenia, mucosal ulcers, or serositis. In these cases, the affected domain only scored 1 or 2 points using SLEDAI-2K, which is less than the 4-point increment in the SLEDAI-2K flare definition or the 3-point increment of c-SFI and r-SFI. 

However, Leosuthamas et al. evaluated the performance of five SLE disease activity indices in a study of 27 patients with active SLE [20]. These indices included the SLE-DAS (Δ ≥ 1.72), SLEDAI-2K responder index-50 (SRI-50), the BILAG-based Composite Lupus Assessment (BICLA), SLE responder index-4 (SRI-4), and a variant replacing SLEDAI-2K with SRI-50 in SRI-4 (denoted as SRI-4(50)). The researchers found no significant differences between these indices when assessing clinical improvement based on PGA and lupus-related medication in patients with SLE. However, the study’s relatively small sample size was a limitation. 

It is worth noting that the BILAG is another important tool for detecting disease activity and treatment response in SLE [21]. The BILAG was initially developed by a group of British and Irish rheumatologists in 1988 [22]. Unlike other scoring systems that rely on a cumulative score to assess disease activity, BILAG uses an alphabetical grading system (A to E) for each organ system to indicate the level of disease activity and the physician’s intention to treat. The revised BILAG index (BILAG 2004) was developed from the original index using a nominal consensus approach by members of BILAG. 

In brief, for each organ or system, a grade A (action) score implies patients require high-dose steroids or immunosuppressive therapy, a grade B (beware) is for those requiring a lower level of immunosuppression, a grade C (contentment) represents low disease activity requiring little treatment, a grade D (discount) means patients had been active in the past but are no longer active, and a grade E (never ever active) means that the disease has never been active in that particular organ or system [23]. 

Despite its utility, the scoring process of BILAG is complex and time-consuming, often requiring specialized training for evaluators. To address this, the Easy-BILAG was developed, building upon BILAG-2004, to provide a more rapid and accurate evaluation across all SLE clinical settings [16]. Easy-BILAG is a single-page document that uses color-coding to indicate disease activity in nine domains. The domains are mucocutaneous, musculoskeletal, cardiorespiratory, neuropsychiatric, hematological, gastrointestinal, ophthalmic, constitutional, and renal. For each domain, Easy-BILAG scores the clinical items as not present, improving, same, worse, or new. Rare manifestations are scored, only when necessary, on a second page. Moreover, Easy-BILAG contains many constitutional items including fever, weight loss, anorexia, lymphadenopathy, and splenomegaly that are more detailed than the SLE-DAS. Furthermore, its sub-classification for individual items is also more detailed. For instance, while SLE-DAS only considers swollen joints, Easy-BILAG also accounts for inflammatory joints in addition to swollen joints. 

In a validation study, Easy-BILAG demonstrated a higher median scoring accuracy (96.7%) compared to the standard BILAG-2004 documentation (87.8%, *p* = 0.001), and exhibited improved inter-rater agreement. Moreover, Easy-BILAG completion was faster, taking a mean of 59.5 min, as opposed to the 80.0 min required for the standard format (*p* = 0.04) when assessing 10 model case vignettes [16]. Although no direct comparisons have been made between BILAG-based scoring systems and SLE-DAS in evaluating SLE disease activity, a recent review of the current state of SLE assessment concluded that both tools represent marked advancements in the assessment of SLE. Easy-BILAG is recognized for its simplicity and error-proof design, making it highly suitable for both clinical trials and routine practice. In contrast, SLE-DAS, although more complex due to its requirement for laboratory parameters and a detailed formula, offers a more detailed and continuous measure of disease activity. This characteristic of SLE-DAS is especially useful in detecting incremental improvements in SLE management [24].

## 6. The Association of SLE-DAS with Health-Related Quality of Life (HRQoL)

Traditionally, the evaluation of disease activity and organ damage in SLE relies on physician assessment. However, there has been a growing recognition of the importance of patient-reported outcomes, particularly regarding the impact of SLE on quality of life (QoL) [25]. Although tools like the Medical Outcomes Study Short Form (SF-36) can be used for assessing HRQoL in patients with SLE, there are several instruments developed specifically for HRQoL in patients with SLE, such as the Lupus Quality of Life (LupusQoL) questionnaire, the SLE-specific Quality of Life (SLEQoL) questionnaire, and the SLE Quality of Life (L-QoL) questionnaire [26]. There are domains that are common to these questionnaires and also those unique to each questionnaire to address specific aspects of life affected by SLE.

For example, the LupusQoL is one of the HRQoL questionnaires designed for SLE [27]. In our study involving 333 Taiwanese patients with SLE, both SLEDAI-2K and SLE-DAS were significantly associated with all eight domains of the LupusQoL. No discernible differences were observed in the correlation coefficients of SLEDAI-2K and SLE-DAS with LupusQoL [18]. Similarly, Onishi et al. showed comparable correlation coefficients between SLE-DAS and SLEDAI-2K in HRQoL as measured by the LupusPRO [28]. In contrast, Ferreira et al. found no significant correlations between the Portuguese version of SLEQoL and SLE-DAS [29,30], while Jiang et al. reported a significant association between SLEQoL and SLEDAI-2K among the Chinese population [31]. 

The variability in HRQoL among SLE patients can be attributed to numerous factors, such as socio-economic status, educational level, medication use, specific clinical manifestations, and coexisting diseases [32]. A meta-analysis incorporating data from 40 studies with 6079 adults with SLE revealed only mild to moderate correlations between disease activity and SF-36 and LupusPRO measurement domains. Notably, SLE disease activity demonstrated strong inverse correlations with SF-36 domains in African and European patients, whereas in Asian populations, organ damage exhibited the strongest inverse correlation with the domains in SF-36 [33]. These discrepancies in the literature suggest that cultural, regional, and methodological differences may influence the reported outcomes. Further research is needed to understand these variations and develop more universally applicable HRQoL measures for patients with SLE.

## 7. SLE-DAS as a Predictor for Hospitalization Risk in SLE

To facilitate its clinical application, SLE disease activity is categorized into four states: severe (SLEDAI-2K > 12), moderate (6 < SLEDAI-2K ≤ 12), mild (0 < SLEDAI-2K ≤ 6), and remission (SLEDAI-2K = 0) [2]. Similarly, SLE-DAS is divided into three states: moderate/severe (SLE-DAS > 7.64), mild (2.08 < SLE-DAS ≤ 7.64), and remission (SLE-DAS ≤ 2.08 and a prednisolone dose ≤ 5 mg/day) [34]. 

In a prospective cohort study involving 326 Taiwanese patients with SLE, individuals categorized as having moderate or severe disease activity according to SLE-DAS were associated with a significantly higher risk of hospital admissions. This increased risk was significant for both SLE-specific and all-cause hospital admissions. In contrast, when the SLEDAI-2K index was used for classification, there was no significant correlation with SLE-specific hospitalizations and only a marginal association with all-cause admissions [35]. Similarly, Wang et al. showed that patients classified with moderate or severe activity by the SLE-DAS had more frequent hospitalizations for both overall and SLE-related issues. Conversely, the moderate or severe activity classification by SLEDAI-2K was only significantly associated with an increase in overall hospital admission rates for patients with SLE [36]. This difference may be explained by the inclusion of cardiac or pulmonary involvement of SLE in SLE-DAS compared to SLEDAI-2K. 

In our previous study [35], we identified three patients with SLE admitted due to pulmonary hypertension and one due to interstitial lung disease. These patients were classified as having moderate or severe status by SLE-DAS but were classified as having only low or moderate disease activity by SLEDAI-2K. As patients with cardiac or pulmonary involvement are known to have a higher risk of hospitalization, SLE-DAS, which accounts for a broader spectrum of symptoms, including cardiac or pulmonary involvement, assigns higher scores to these conditions. Consequently, it shows a higher risk for hospital admission. In contrast, the SLEDAI-2K may not reflect the severity of these specific organ involvements to the same extent, potentially underestimating the associated risk. This suggests that SLE-DAS may provide a more sensitive measure of disease activity that aligns closely with the need for hospital-based care.

## 8. Assessing SLE Disease Activity during Pregnancy Using SLE-DAS

Pregnancy introduces maternal physiological changes in patients with SLE, complicating disease activity assessments. Symptoms like edema, proteinuria, or hypertension might be attributed to either normal pregnancy changes or lupus flares. Buyon et al. introduced a modified version of SLEDAI-2K, termed the SLE-pregnancy disease activity index (SLEPDAI) [37]. However, SLEPDAI has not yet been fully validated, and further studies are needed to confirm its reliability and accuracy. 

Larosa et al. evaluated SLE-DAS and SLEPDAI in the first trimester to predict maternal flare-ups and obstetric complications in the second and third trimesters among a cohort of 158 pregnant women with SLE. A high correlation between SLE-DAS and SLEPDAI in the first trimester was observed (ρ = 0.97, *p* < 0.01). Both SLE-DAS and SLEPDAI were found to be predictive of maternal flares associated with adverse pregnancy outcomes (APOs), including fetal and neonatal mortality, premature delivery before 37 weeks due to placental insufficiency, and birth of infants small for their gestational age. Moreover, the SLE-DAS model performed slightly better than SLEPDAI model, as assessed by the area under the receiver operating characteristic curve and goodness-of-fit analysis [38]. While both SLEPDAI and SLE-DAS are simple and effective in predicting maternal flares and adverse pregnancy outcomes, SLE-DAS might be favored for its potential ease of use and detailed continuous assessment capabilities, which are important in the dynamic context of managing SLE during pregnancy.

## 9. SLE-DAS as a Treatment Target for SLE

The treat-to-target (T2T) strategy has emerged as a proposed approach for SLE management, with low disease activity (LDA) or remission status as the predefined targets. The lupus low disease activity state (LLDAS) framework, introduced in 2016 [39], mandates not only a SLEDAI-2K score of ≤ 4 but also the absence of new disease activity in major organ systems, a SELENA-SLEDAI PGA score (ranging from 0–3) of ≤ 1, low doses of steroids, and stable use of immunosuppressive or biological agents. Achieving LLDAS has been associated with a significant reduction in organ damage risks and, most importantly, a lower mortality risk [40,41]. Consequently, many clinical trials have adopted LLDAS as a pivotal outcome measurement [42,43]. 

However, measuring LLDAS in routine clinical settings can be challenging. Abdelhady et al. showed a good agreement between SLEDAI-2K-derived LLDAS and SLE-DAS in a study of 117 patients with SLE [44]. Assunção et al. found that SLE-DAS LDA (defined as SLE-DAS ≤ 2.48 and a daily prednisone dose or its equivalent ≤ 7.5 mg) correlated well with SLEDAI-2K-derived LLDAS in a study involving 774 patients with SLE, with 300 in the derivation cohort and 474 in the validation cohort [45]. They found that a small proportion of patients exhibiting active lupus arthritis (1.03%), skin rashes (1.37%), and mucosal ulcers (0.34%) still qualified for LLDAS. However, none with these symptoms were classified as SLE-DAS LDA subgroups. This suggests that SLE-DAS LDA might offer a more apparent target in T2T strategies. In addition, Cunha et al. noted that 7.5% of patients with SLE meeting the LLDAS criteria did not achieve SLE-DAS LDA, with a higher SLE-DAS score at baseline predicting potential flare-ups [46]. The comparative value of SLE-DAS LDA and LLDAS in predicting SLE flare-ups remains to be elucidated.

Another T2T in SLE treatment is achieving remission. In 2021, the Definitions Of Remission In SLE (DORIS) Initiative outlined SLE remission using the following criteria: a clinical SLEDAI score of 0, an evaluator’s global assessment of < 0.5 (on a scale of 0–3), a daily prednisolone dose of 5 mg or less, and stable doses of antimalarials, immunosuppressants, and biologics [47]. If a patient meets all of the DORIS criteria, they are considered to be in remission. Based on SLE-DAS, two remission criteria have been proposed. The first, termed SLE-DAS index-based clinical remission, requires an SLE-DAS score of ≤ 2.08 and a daily prednisone dosage of ≤ 5 mg. The second, known as Boolean-based clinical remission, mandates a score of 0 in all clinical items of SLE-DAS along with a daily prednisone dosage of ≤ 5 mg [34]. The key difference between these definitions lies in the Boolean definition’s requirement for specific low thresholds in each component, making it more rigid. Patients must meet all criteria at a single point in time to be considered in remission. On the other hand, the index-based definition provides a cumulative score reflecting overall disease activity, allowing some flexibility in individual components.

Originally, SLE-DAS was developed with a primary focus to assess disease activity in SLE patients, which inherently includes implications for evaluating treatment response [15]. Its utility was later expanded to effectively define SLE clinical remission states and disease activity categories, with its effectiveness validated against expert assessment and the BILAG index. Furthermore, both SLE-DAS remission criteria have been found to be comparable to the DORIS clinical remission criteria. An added advantage of SLE-DAS is its ease of use; it does not require an evaluator’s global assessment or specific medication stipulations regarding antimalarials, immunosuppressives, and biologics, unlike the DORIS criteria [33]. Moreover, SLE-DAS offers two distinct approaches—index-based and Boolean-based—for assessing remission. This approach provides flexibility, potentially making SLE-DAS more adaptable to different clinical scenarios compared to the more rigid DORIS criteria. A summary of recent studies comparing SLE-DAS to other SLE-related indices is shown in Table 1.

## 10. Limitations and Future Perspectives

While SLE-DAS shows promising potential, several aspects of its utility require further exploration. First, patients with SLE represent a wide spectrum of demographics, including variations in ethnicity, age, and sex. These diverse groups may exhibit distinct clinical manifestations of the disease. Currently, there is insufficient data to robustly validate the effectiveness of SLE-DAS across these subgroups. Consequently, there is a need for more extensive and diverse data collection and analysis to demonstrate the applicability of SLE-DAS across the entire spectrum of SLE.

Second, research by Jesus et al. demonstrated that both SLE-DAS and SLEDAI-2K could predict SLE-related organ damage, as defined by the SDI. The SDI is designed specifically to quantify the cumulative, non-reversible damage in patients with SLE over time resulting from disease and its treatment. However, the lack of direct comparative data between SLE-DAS and SLEDAI-2K indicates that more comprehensive evidence is required to establish the superiority of SLE-DAS over SLEDAI-2K in predicting organ damage [15]. 

Third, a retrospective study of 41 patients with lupus nephritis indicated that SLE-DAS showed a stronger correlation with SLEDAI-2K when the disease activity was low. However, its performance in cases with high disease activity was less certain. The researchers concluded that further validation is needed for the performance of SLE-DAS in patients with high disease activity, particularly those exhibiting active urine sediments but not having proteinuria [17]. In response to this study, Jesus et al. explained that the inclusion of urinary sediment in disease activity measures has limitations due to technical issues, a lack of specificity, and its association with both activity and chronicity indexes in renal biopsies. They also noted that persistent isolated microscopic hematuria in lupus nephritis has not been associated with negative outcomes [48].

Fourth, a direct comparison between Easy-BILAG and SLE-DAS is still lacking [49]. The inclusion of cardiac and pulmonary involvement in SLE-DAS is a notable improvement, given their known association with higher mortality rates in patients with SLE [50,51]. However, SLE-DAS does not list several rare but severe SLE clinical manifestations, including ophthalmic involvement, panniculitis/bullae in skin, and gastrointestinal involvement. The impact of these omissions on the effectiveness of SLE-DAS warrants additional research. In comparison with Easy-BILAG, SLE-DAS has far fewer items that need to be evaluated. Future prospective studies should focus on establishing a clear correlation between SLE-DAS and Easy-BILAG and determine their respective abilities to assess the risk of organ damage and mortality in patients with SLE.

Finally, it is noteworthy that cardiac involvement and hemolytic anemia are more common in men with SLE than in women [52,53]. The inclusion of these conditions in SLE-DAS might be especially beneficial for the male population. Future research that focuses on sex differences in the performance of SLE-DAS would be invaluable to further understand and improve the management of SLE. 

## 11. Conclusions

The advent of the SLE-DAS and its disease activity classification presents a potentially invaluable tool for rheumatologists, particularly for its utility in effectively assessing clinical responses, predicting hospitalizations, and guiding treatment objectives in patients with SLE. As efforts continue to refine disease activity measures, tools like SLE-DAS become pivotal in enhancing the management of SLE. While further research and comparative studies are essential to fully establish its superiority and applicability, the current evidence underscores the promise and relevance of SLE-DAS in contemporary clinical practice. Ultimately, this tool is expected to contribute to improved patient outcomes.

## Figures and Tables

**Table 1:** Recent studies comparing Systemic Lupus Erythematosus Disease Activity Score (SLE-DAS) to other SLE-related indices.

Goal	Index Compared	Results	Number of Patients (Ethnicity)	Reference Number
Clinically meaningful changes	ΔSLE-DAS ≥ 1.72 and ΔSLEDAI-2K ≥ 4	ΔSLE-DAS ≥ 1.72 sensitivity 82.1%, specificity 96.9%; ΔSLEDAI-2K ≥ 4 sensitivity 44.8%, specificity 96.5%; *p* < 0.0005.	196 (validation cohort) (94.4% Caucasian)	[15]
Identification of flare	ΔSLE-DAS ≥ 1.72, classic-SELENA Flare Index (c-SFI), revised-SFI (r-SFI), and SLEDAI-2K (score increase ≥ 4)	Sensitivity: SLE-DAS 97.1%, c-SFI 88.4%, r-SFI 88.4%, SLEDAI-2K 56.5%. Specificity: SLE-DAS 97.3%, c-SFI 98.1%, r-SFI 96.8%, SLEDAI-2K 99.2%. Result of ROC analysis: ΔSLE-DAS ≥ 1.72 is best.	442 (95.7% Caucasian)	[19]
Classified responders vs. non-responders	SLEDAI-2K responder index-50 (SRI-50), SLE responder index-4 (SRI-4), substituting SLEDAI-2K with SRI-50 in SRI-4 (SRI-4(50)), ΔSLE-DAS ≥ 1.72, and BICLA	No differences.	27 (Asian)	[20]
Association with health-related quality of life (LupusQoL)	SLEDAI-2K and SLE-DAS	No differences.	333 (Asian)	[18]
Association with health-related quality of life (LupusPRO)	SLEDAI-2K and SLE-DAS	No differences.	335 (Asian)	[28]
Predict risk of hospitalization	SLE disease activity classified by SLEDAI-2K and SLE-DAS	SLE-DAS, but not SLEDAI-2K, was associated with an increased risk for all-cause and SLE-related hospitalization.	326 (Asian)	[35]
Predict frequency of hospitalization	SLE disease activity classified by SLEDAI-2K and SLE-DAS	Both SLE-DAS and SLEDAI-2K were associated with frequency of all-cause hospitalization. SLE-DAS, but not SLEDAI-2K, was associated with frequency of SLE-related hospitalization.	324 (Asian)	[36]
Predict maternal flare	SLE-DAS and SLE-pregnancy disease activity index (SLEPDAI)	Both SLE-DAS and SLEPDAI predicted maternal flares and adverse pregnancy outcomes.	158 (White 78.5%, Black 13.3%, Asian 6.9%)	[38]
Definitions of clinical remission	SLE-DAS Boolean-based remission criteria, SLE-DAS index-based remission criteria, and DORIS definition of remission	Both the SLE-DAS Boolean-based remission criteria and SLE-DAS index-based remission criteria were comparable with the DORIS clinical remission criteria.	969 (validation cohorts) (White 67.4%, Black 15.5%, Hispanic 18.3%)	[34]

ΔSLE-DAS: change in Systemic Lupus Erythematosus Disease Activity Score. ΔSLEDAI-2K: change in Systemic Lupus Erythematosus Disease Activity Index 2000. BICLA: British Isles Lupus Assessment Group (BILAG)-based Composite Lupus Assessment. DORIS: Definitions Of Remission In SLE. LupusQoL: Lupus Quality of Life. LupusPRO: Lupus Patient-Reported Outcome tool. SELENA: Safety of Estrogens in Lupus Erythematosus National Assessment. ROC: receiver operating characteristic curve. Adverse pregnancy outcomes include fetal and neonatal death, placental insufficiency with premature delivery < 37 weeks, and small for gestational age infants.

## Data Availability

No new data were created or analyzed in this study. Data sharing is not applicable to this article.
